# Delayed Onset Hypoparathyroidism Following Thyroidectomy: Case Report and Review of Literature

**DOI:** 10.7759/cureus.103665

**Published:** 2026-02-15

**Authors:** Sonam Tshering, Swetha Kotha, Nay Chi Kyaw, Animesh Gupta, Ashutosh Kapoor

**Affiliations:** 1 Endocrinology and Diabetes, Barts Health NHS Trust, London, GBR; 2 Endocrinology and Diabetes, Barking, Havering and Redbridge University Hospitals NHS Trust, London, GBR; 3 Diabetes and Endocrinology, Barts Health NHS Trust, London, GBR; 4 Acute Internal Medicine, London North West University Healthcare NHS Trust, London, GBR

**Keywords:** chronic hypoparathyroidism, delayed hypoparathyroidism, hypocalcemia, post-surgical hypoparathyroidism, thyroidectomy complications

## Abstract

Delayed postsurgical hypoparathyroidism (hypoPT) is a rare but important complication of thyroidectomy, often presenting years after the initial procedure. We report the case of a 48-year-old woman with a remote history of total thyroidectomy for Graves’ disease, who presented with neuromuscular symptoms and severe hypocalcemia 22 years after surgery. Laboratory investigations revealed low calcium, low parathyroid hormone (PTH), and elevated phosphate, consistent with hypoPT. The patient was initially managed with intravenous calcium followed by alfacalcidol therapy. Literature review highlights that most cases of delayed hypoPT occur in women following total thyroidectomy and vitamin D deficiency. Mechanisms may involve progressive parathyroid hypovascularisation and scar retraction. Current guidelines do not recommend routine long-term calcium monitoring post thyroidectomy, but this case underscores the need for vigilance and patient education regarding hypocalcemia symptoms. Lifelong follow-up may be warranted for high-risk individuals.

## Introduction

Chronic hypoparathyroidism (hypoPT) is a rare condition characterized by hypocalcemia, hyperphosphatemia, and absent or deﬁcient production of parathyroid hormone (PTH) [1]. Its prevalence ranges from 6.4 to 37 per 100,000 people, with an incidence of 0.8 to 2.6 per 100,000 annually [1]. Postsurgical HypoPT constitutes approximately 75% of all cases [2]. Transient postsurgical hypoPT occurs in 5.4% to 27.9% of cases, while permanent postsurgical hypoPT occurs in 0.3% to 16.7% [1].

The mechanisms of postsurgical hypoPT include disruption of blood flow to the parathyroid glands, mechanical trauma, thermal or electrical injury, and either deliberate or accidental partial or complete removal of the glands [3].

Clinically, hypoPT leads to hypocalcemia, which may present as perioral and fingertip tingling, muscle cramps, and spasms [4]. Neuropsychiatric symptoms such as mood changes, confusion, and dizziness can also occur [4]. Severe hypocalcemia may cause laryngospasm or seizures. Clinical signs include tetany, a positive Chvostek’s or Trousseau’s sign, and, in advanced cases, cardiac arrythmias like QT prolongation and torsades de pointes [4].

Common complications of chronic hypoPT include cataracts (median prevalence 17%), nephrocalcinosis or kidney stones (15%), renal insufficiency (12%), infections (11%), seizures (11%), depression (9%), and ischemic heart disease (7%) [2].

Following thyroidectomy, 70-80% of patients with parathyroid dysfunction recover within a month [2]. HypoPT is considered permanent if it persists for more than a year after surgery [2]. Measuring PTH levels within 12-24 hours post total thyroidectomy is recommended; patients with PTH above 10 pg/mL (1.05 pmol/L) during this period are unlikely to develop permanent hypoPT [2].

A retrospective cohort study demonstrated that the incidence of hypoPT increases over time following surgery, starting at 10.1% upon discharge and reaching 12.6% after more than a year, with a median follow-up of nearly eight years [5]. The risk of persistent hypoPT was significantly greater in patients who underwent re-operation for bleeding or had surgery for thyroid cancer [5]. These results highlight the need for ongoing monitoring of parathyroid function after surgery to detect both delayed onset and late recovery of hypoPT [5].

We describe a case of hypoPT that appeared long after surgery and have reviewed the literature to address the limited knowledge surrounding this condition.

## Case presentation

A 48‑year‑old woman was referred to the emergency department following the identification of severe hypocalcemia in the community, with an adjusted calcium level of 1.7 mmol/L. She reported several months of intermittent tingling sensations in both legs, accompanied by lethargy. Her past medical history included total thyroidectomy for Graves’ disease 22 years ago outside the United Kingdom, resulting in postsurgical hypothyroidism. She also had a history of hypertension and osteoarthritis. Her regular medications were levothyroxine 100 mcg once daily and candesartan 16 mg once daily. She was unaware of any low calcium issues previously; however, we did not have any baseline laboratory investigations to compare as she was new to our service. She did not report any history of low calcium in her family members.

On examination, she demonstrated a positive Chvostek’s sign. Her vital signs were stable, with an SpO₂ of 99% on room air, a respiratory rate of 17 breaths per minute, a heart rate of 82 beats per minute, and a temperature of 37.5°C. Her blood pressure was elevated at 158/97 mmHg. Electrocardiography showed normal sinus rhythm with no arrhythmias and normal QTc interval.

Laboratory investigations revealed hypocalcemia, hyperphosphatemia and low PTH levels consistent with hypoPT (Table 1). Despite vitamin D deficiency, PTH level was low indicating true hypoPT. Serum magnesium was within normal range ruling out functional hypoPT.

**Table 1:** Laboratory investigations on admission and at follow up PTH: Parathyroid hormone; TSH: Thyroid-stimulating hormone; HbA1c: Glycated hemoglobin; IgA: Immunoglobulin A

Parameter	On admission	Follow up at 6 months (on alfacalcidol 1 mcg once daily)	Reference range
Adjusted calcium serum	1.71	2.24	2.2-2.6 mmol/L
Inorganic phosphate serum	1.92	1.09	0.8-1.51 mmol/L
PTH	1.2	N/A	1.8-7.9 pmol/L
25-OH vitamin D	26	N/A	<25 nmol/L is deficient
Magnesium serum	0.9	N/A	0.7-1 mmol/L
Sodium serum	141	142	133-146 mmol/L
Potassium serum	4.4	4	3.5-5.3 mmol/L
Creatinine serum	79	93	45-84 umol/L
Alkaline phosphatase serum	90	56	30-130 unit/L
Albumin Serum	45	47	35-50 g/L
Free T4 serum	18	24.5	10.5-24.5 pmol/L
TSH	6.58	1.55	0.27-4.2 Mu/L
Vitamin B12 serum	357	N/A	197-1,000 ng/L
Folate serum	8.7	N/A	3.9-9.9 mcg/L
HbA1c	32	N/A	<42 mmol/mol
Tissue transglutaminase IgA antibody	1	N/A	0-5 kunits/L

There was no evidence of fungal infections, and celiac disease testing (anti-tissue transglutaminase antibodies) returned negative results. Additionally, she did not display any clinical or laboratory signs indicative of Addison’s disease. Therefore, an autoimmune cause for hypoPT was not supported, and considering her previous thyroid surgery, a diagnosis of postsurgical hypoPT was established. 

Initial treatment included intravenous calcium gluconate infusion and she was started on alfacalcidol at 1 mcg daily. She was also given a loading dose of colecalciferol 100,000 units once a day for two days for vitamin D insufficiency, followed by 1,000 units per day.

On follow up at six months, she remains on alfacalcidol 1 mcg once a day and remained symptom-free and eucalcemic (adjusted serum calcium of 2.24 mmol/L, serum phosphate of 1.09 mmol/L).

## Discussion

Delayed diagnosis and mechanisms

The diagnosis of postsurgical hypoPT can be missed or delayed, particularly when patients do not recognize the symptoms of hypocalcemia [6]. Clinicians should therefore consider measuring serum calcium in individuals who present with neuromuscular irritability, cognitive disturbances, or who are at increased genetic risk [6]. Delayed onset of hypoPT, occurring several years after surgery, is rare and its underlying mechanisms are not fully understood. Proposed explanations include scar retraction of parathyroids and progressive reduction in blood supply to parathyroid glands that were already compromised during the initial operation [7].

Epidemiology and risk factors

A retrospective review of 406 patients with well-differentiated thyroid cancer who underwent total thyroidectomy found that 6.16% developed delayed-onset mild hypoPT at least one-year post surgery, despite initially normal calcium levels [8]. Notably, 76% of these cases had received radioactive iodine therapy, suggesting a potential association [8]. Meta-analyses have identified several risk factors for both transient and permanent hypoPT, including female sex, advanced nodal stage, central and lateral neck dissection, bilateral central neck dissection, total thyroidectomy (compared to lobectomy or sub-total thyroidectomy), incidental parathyroidectomy, and malignant pathology [9]. Node metastasis and the presence of parathyroid tissue in the surgical specimen are specifically linked to permanent hypoPT [9]. Low preoperative calcium and vitamin D levels are common and independently increase the risk of both transient and permanent hypoPT, with severe vitamin D deficiency particularly raising the likelihood of transient hypocalcemia and, to a lesser extent, permanent hypoPT [10]. Additional predictors include total thyroidectomy, removal of level VI lymph nodes, retention of fewer than two parathyroid glands, repeat surgery, surgical technique variations, and a diagnosis of Graves’ disease [11]. Younger age (especially in pediatric patients), female sex, pregnancy, and vitamin D deficiency are also associated with a higher risk of postoperative hypocalcemia [11].

Case series and clinical features

A review of published case reports on delayed hypoPT after surgery shows that most affected individuals were women (nine out of 10 cases), all of whom had undergone total thyroidectomy (Table 2). The time between surgery and the onset of hypoPT varied widely, with some cases appearing as late as 36 years post operation. The majority of patients had 25-OH vitamin D levels near the lower limit of normal, and two were found to be deficient. Magnesium levels were within normal limits in seven out of 10 cases, while three cases either lacked magnesium measurements or did not mention them, effectively excluding functional hypoPT. None of the patients had concurrent autoimmune disorders. These observations align with known risk factors for permanent hypoPT. Notably, four out of 10 patients experienced seizures, underscoring the potentially serious nature of delayed presentation.

**Table 2:** Series of case reports with delayed presentation of postsurgical hypoPT hypoPT: Hypoparathyroidism

Case reports	Age	Gender	Time to presentation (years)	Type of surgery	25-OH vitamin D levels	Magnesium levels	Predominant clinical features
Kamath et al. [12]	76	Female	25	Total thyroidectomy for goitre	27 ng/ml (>30 ng/ml is sufficient)	N/A	Seizures, basal ganglia calcification, prolonged QTc interval
Simões et al. [7]	24	Female	3.5	Total thyroidectomy for Grave’s disease	32 ng/ml (>30 ng/ml is sufficient)	N/A	Muscle cramps
Wijewickrama et al. [13]	60	Female	30	Total thyroidectomy for follicular thyroid carcinoma	32 ng/ml (>30 ng/ml is sufficient)	0.9 mmol/L (Ref 0.85-1.1 mmol/L)	Muscle cramps, perioral and fingertip numbness
	53	Female	12	Total thyroidectomy for papillary thyroid carcinoma	38 ng/ml (Ref 30-100 ng/ml)	0.9 mmol/L (Ref 0.85-1.1 mmol/L)	Muscle cramps, pins and needles
	49	Female	11	Completion thyroidectomy for incidental papillary thyroid carcinoma	33 ng/ml (>30 ng/ml is sufficient)	1.4 meq/L (Ref 1.3-2.1 meq/L)	Muscle cramps
Abbas et al. [14]	26	Female	8	Total thyroidectomy for goiter	30.1 ng/ml (>30 ng/ml is sufficient)	2.2 mg/dl (normal)	Joint pains, muscle spasms
Mashadi et al. [15]	70	Female	12	Total thyroidectomy for multinodular goiter	57 nmol/L (Ref 50-120 nmol/L)	0.85 mmol/L (0.7-1 mmol/L)	Tinnitus and tingling in her fingertips
Garg et al. [16]	42	Male	19	Total thyroidectomy for papillary thyroid carcinoma	N/A	N/A	Seizure
Hamid et al. [17]	52	Female	25	Total thyroidectomy for papillary thyroid carcinoma	19 nmol/L (< 25 nmol/L is deficient)	0.72 mmol/L (Ref 0.7-1 mmol/L)	Seizure
Bellamy et al. [18]	47	Female	36	Total thyroidectomy for papillary thyroid carcinoma	N/A	0.69 mmol/L (Ref 0.7-1 mmol/L)	Joint pains, muscle aches

Genetic testing recommendations

Summary statement and guidelines from the second international workshop recommends considering genetic testing for individuals with nonsurgical hypoPT who have a family history of the condition, exhibit features of an associated syndrome, or are younger than 40 years [2]. For those with clinical features suggestive of autoimmune polyendocrinopathy-candidiasis-ectodermal dystrophy syndrome (APECED), testing for variants in the autoimmune regulator (AIRE) gene is advised [2]. In the present case, these criteria were not met due to the history of surgery, age over 40, lack of family history, and absence of autoimmune features. Conditions such as pseudo-hypoPT or vitamin D deficiency can also cause hypocalcemia, but these are typically characterized by elevated PTH levels [2].

Sex differences and hormonal influences

Women are at increased risk of both transient and permanent hypoPT due to several factors [9]. They often present with more severe thyroid conditions before surgery, such as hyperthyroidism or thyromegaly, which raises the chance of parathyroid injury [9]. Differences in hormone and vitamin D levels between sexes also influence postoperative parathyroid recovery [9]. Estrogen influences parathyroid function and enhances calcium absorption from the intestines [9]. Hormonal changes, especially during menopause, and emotional responses to illness and surgery may further disrupt hormone balance, increasing risk [9]. Additionally, stronger immune responses in women can affect postoperative inflammation and healing, potentially leading to parathyroid dysfunction [9].

Management of postsurgical hypoPT

Both the Second International Workshop and the American Thyroid Association recommend standard treatment for postsurgical hypoPT with oral calcium and active vitamin D [2,4]. Colecalciferol is also advised to maintain normal 25-OH vitamin D levels [2]. If conventional therapies do not adequately control the condition, PTH therapy may be considered [2,4]. The primary goal in managing hypoPT over the long term is to keep serum calcium levels within a range that prevents symptoms, while also avoiding both significant hypocalcemia and hypercalcemia and their related complications [4]. Preserving bone health is also a key objective [4]. To reduce the risk of symptoms, it is recommended that serum calcium be maintained at the lower end of the normal range, and serum phosphorus should not exceed the upper limit of normal [4]. Recommendations include maintaining 24-hour urinary calcium excretion below 7.5 mmol/day and keeping the calcium-phosphorus product under 55 mg²/dL² [4].

Monitoring and patient education

At present, there are no official recommendations for ongoing calcium monitoring in patients who have normal calcium levels shortly after undergoing total thyroidectomy. Nevertheless, evidence from this case and the wider literature indicates that those at increased risk should be informed about the symptoms of hypocalcemia and may benefit from periodic calcium assessments to help prevent complications arising from delayed hypocalcemia.

## Conclusions

Delayed postsurgical hypoPT is an uncommon but clinically significant complication that can arise many years after thyroidectomy. This case and literature review demonstrates that patients with a history of total thyroidectomy, especially females and those with additional risk factors, such as vitamin D deficiency, may develop hypoPT long after their initial surgery. Early recognition of symptoms and prompt management with calcium and active vitamin D analogues are crucial for favorable long-term outcomes. Although routine long-term calcium monitoring is not currently recommended for all post-thyroidectomy patients, this case highlights the importance of patient education and vigilance, particularly for high-risk individuals. Lifelong follow-up and periodic assessment for high-risk patients may be warranted to prevent complications from delayed hypocalcemia.
